# Transition between protein-like and polymer-like dynamic behavior: Internal friction in unfolded apomyoglobin depends on denaturing conditions

**DOI:** 10.1038/s41598-020-57775-4

**Published:** 2020-01-31

**Authors:** Livia Balacescu, Tobias E. Schrader, Aurel Radulescu, Piotr Zolnierczuk, Olaf Holderer, Stefano Pasini, Jörg Fitter, Andreas M. Stadler

**Affiliations:** 1 Forschungszentrum Jülich GmbH, Jülich Centre for Neutron Science (JCNS) at Heinz Maier-Leibnitz Zentrum (MLZ), Lichtenbergstr. 1, 85748 Garching, Germany; 2 I. Physikalisches Institut (IA), AG Biophysik, RWTH Aachen, Germany; 3 Forschungszentrum Jülich GmbH, Jülich Centre for Neutron Science (JCNS) Outstation at Spallation Neutron Source (SNS), Oak Ridge, TN 37831 USA; 4 Forschungszentrum Jülich GmbH, Institute for Complex Systems (ICS-5), 52425 Jülich, Germany; 5 Institute of Physical Chemistry, RWTH Aachen University, Landoltweg 2, 52056 Aachen, Germany; 6 Forschungszentrum Jülich GmbH, Jülich Centre for Neutron Science (JCNS-1) and Institute for Complex Systems (ICS-1), 52425 Jülich, Germany

**Keywords:** Protein folding, Protein aggregation, Molecular conformation, SAXS, Biological physics

## Abstract

Equilibrium dynamics of different folding intermediates and denatured states is strongly connected to the exploration of the conformational space on the nanosecond time scale and might have implications in understanding protein folding. For the first time, the same protein system apomyoglobin has been investigated using neutron spin-echo spectroscopy in different states: native-like, partially folded (molten globule) and completely unfolded, following two different unfolding paths: using acid or guanidinium chloride (GdmCl). While the internal dynamics of the native-like state can be understood using normal mode analysis based on high resolution structural information of myoglobin, for the unfolded and even for the molten globule states, models from polymer science are employed. The Zimm model accurately describes the slowly-relaxing, expanded GdmCl-denaturated state, ignoring the individuality of the different aminoacid side chain. The dynamics of the acid unfolded and molten globule state are similar in the framework of the Zimm model with internal friction, where the chains still interact and hinder each other: the first Zimm relaxation time is as large as the internal friction time. Transient formation of secondary structure elements in the acid unfolded and presence of *α*-helices in the molten globule state lead to internal friction to a similar extent.

## Introduction

**Figure 1:**
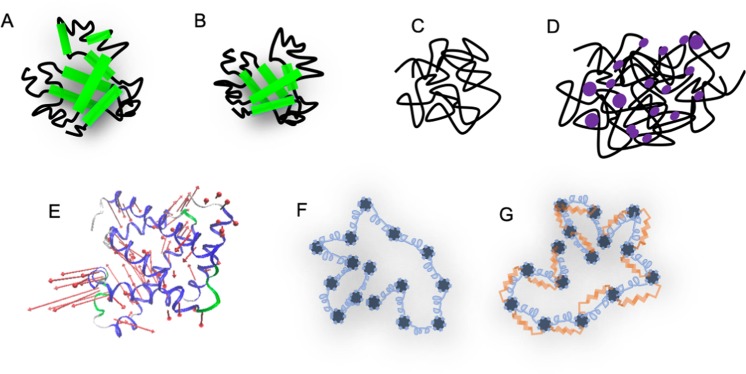
(**A**–**D**) Schematic representations of the different folding states of apoMb: apoMb at pD 6 ((**A**) the 8 *α*-helices are marked with green), apoMb at pD 4 (**B**), apoMb at pD 2 (**C**), apoMb denaturated by GdmCl ((**D**) GdmCl are marked with purple). (**E**) Cartoon representation of the first non-trivial mode of myoglobin 2v1k.pdb. Loops are marked with green and grey, *α*-helices with blue, and the motion vectors with red arrows. (**F**,**G**) Sketches of a polymer interpreted by the Zimm (**F**) and ZIF (**G**) model: the beads are represented by the grey bullets and are interconnected though the springs (blue); additionally, in the ZIF model, the interchain friction is represented through the red dashpot. Each bead interacts with the solvent.

The similarities between polymers and unfolded or disordered proteins behaving like polymers are subject of recent debate. On one hand, the soft matter community brings detailed insights in the physical properties of soft matter macromolecules based on polymer models, including very complex interactions between their simple chemical monomer units. On the other hand, there are biological macromolecules, for example, proteins, which consist of 20 different monomers, whose distinct sequence results in a unique three-dimensional fold and gives rise to a very specialized biological function^1^. An emerging biological interest concerns the dynamical properties of intrinsically unfolded proteins and the relevance of molecular dynamics for the protein folding problem where an unfolded and flexible protein progressively gains secondary structure and finally forms its folded and comparatively rigid structure. In the present manuscript, we studied different folding states of apomyoglobin (apoMb), in order to investigate the transition between protein-like and polymer-like dynamic behavior. ApoMb is a model system for protein folding studies and the heme-free form of myoglobin, a protein responsible for the transport and storage of oxygen in the horse heart muscle (uniprot no. P68082). It consists of 153 amino acids, which form 8 *α*-helices connected by loops. The kinetics of its acid unfolding and folding process are well-characterized^2^: at pH 6 the protein is in a native-alike state, at pH 4 in a molten globule state, whilst at pH 2 the protein is completely unfolded. An alternative denaturation/unfolding path, which has different physical processes lying behind, is induced by adding guanidine hydrochloride (GdmCl). Early studies showed that based on pH and temperature, the GdmCl concentration needed to denaturate myoglobin varies^3^. For the current experiment, the used concentration of GdmCl was 3 M. A schematic representation of these folding states is available in Fig. 1A–D. The nanosecond and nanometer time- and length-regime is of significant relevance in understanding the equilibrium dynamics of unfolded peptide chains. These motions are essential in the process of protein folding as initial steps of protein folding occur in that time-space range. The conformational motions of the unfolded peptide drive the sampling of the energy landscape and the exploration of a large conformational space prior to the collapse into the folded structure. It is currently an experimental challenge to probe these large-scale motions. There are only a few experimental techniques that access these length and timescales, i.e. NMR^4,5^, single-molecule Förster resonance energy transfer combined with nanosecond correlation spectroscopy^6,7^, and neutron spin-echo spectroscopy (NSE)^8,9^. In this regime, several dynamic processes occur: translational and rotational diffusion of the whole molecule which are related to the protein-solvent interactions, and motions within the molecule, e.g. backbone torsional re-orientations, side-chain translations, motions that we call internal dynamics. These motions are driven by the thermal forces from the collision of the protein with solvent molecules (Brownian motion)^10^. Side-chain rotations and local vibrational motions occur on the picosecond time-scale and may lead to coupled motions, and are mostly subject of laser spectroscopy investigations and of quasi-elastic neutron scattering (QENS)^11^.

So far, NSE was employed to investigate and characterize domain dynamics of multidomain proteins such as phosphoglycerate kinase (45–65 kDa)^12^, alcohol dehydrogenase (141 kDa)^13^, immunoglobulin G1 (150 kDa)^14,15^ and Taq-polymerase^16^ (94 kDa), of dimers e.g. bovine serum albumin^17^ (132 kDa), and of smaller proteins such as human lactoferrin (78 kDa)^18^ and the mammalian Na^+^/H^+^ exchange regulatory factor 1 (NHERF1, 50 kDa)^19^. By decoupling the translational-rotational diffusion of the whole system, the NSE spectra deliver information on the internal dynamics, interpreted using normal mode analysis. Other particular applications for this technique are intrinsically disordered and unfolded proteins, which lack secondary structure elements and contain a high degree of structural freedom and conformational flexibility. NSE studies were performed and reported on myelin basic protein^20^, guanidinium- denatured bovine serum albumin^21^. The dynamics of these proteins and their interactions with the solvent were interpreted using three different models: the Zimm model, Zimm with internal friction and Zimm with damping of the mode amplitudes, which are well-established in polymer theory^22,23^. These models have been derived from the Rouse models: Rouse with internal friction^24^ and Rouse with damping of the mode amplitudes^25^. Further variations of the Zimm model, which consider loop formations along the protein chain, were applied to data on intrinsically disordered proteins studied by single-molecule photo-induced energy transfer spectroscopy. By placing a fluorescent label at the end of the protein chain and allowing it to transfer energy to certain amino-acids, one can measure the relaxation of several loop forming chain segments^26–28^. By investigating different folding and denaturation intermediates of apomyoglobin (apoMb), we intend to see to which extent these approaches towards modeling protein dynamics can be applied. This is especially interesting since it will give insight into the regime where proteins behave like polymers, losing the individuality of their different amino acid monomers.

## Results

### Structural properties

**Figure 2:**
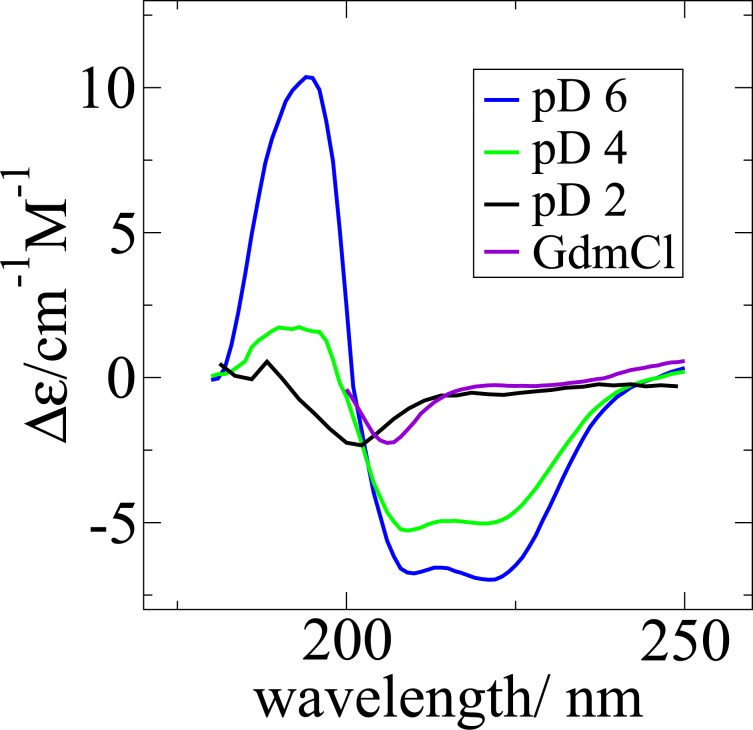
Measured CD spectra of apoMb in different folding states. The curve of apoMb at pD 6 has the typical profile of a globular protein with a significant content of helical structure. The curves of apoMb at pD 2 and in 3 M GdmCl are typical random coils, unfolded states. The spectrum of pD 4 validates the partially folded state of apoMb.

According to the circular dichroism (CD) measurements, apoMb in its native-alike form at pD 6 contains 49% secondary structure elements (see Fig. 2). Under acid denaturation, apoMb at pD 4 has 25% secondary structure elements and at pD 2 4.3%. At 3 M GdmCl, there are about 6% secondary structure elements in the protein molecule. The content of secondary structure elements of apoMb at pD 2 and the one of GdmCl-denatured states are in both cases very small and comparable to each other within the error of the applied technique.

**Figure 3:**
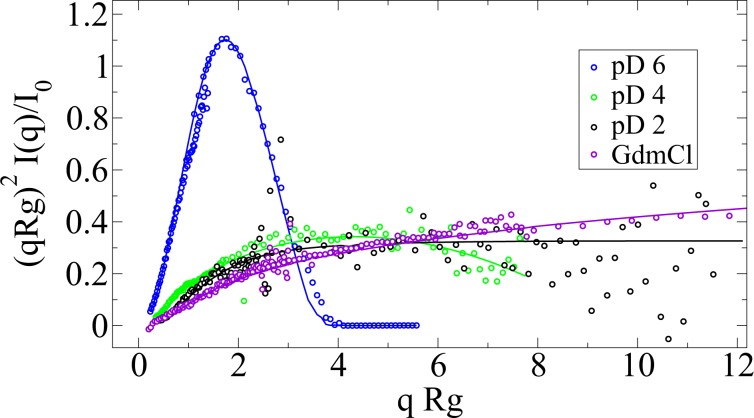
Normalized Kratky-Porod representation of the SANS data with the models used to obtain the form factor. The apoMb at pD 6 structure shows the characteristic peak of a globular protein, the pD 4 is a typical molten globule (reaching a maximum at *qR*_*g*_ = 0.2 Å^−1^ ⋅ 25. 4 Å = 5). pD 2 and GdmCl data are specific for unfolded states.

Small angle neutron scattering (SANS) was used to gain information on these folding states. The labile protons of the protein have been exchanged with deuterium and all the solvents used were deuterated to decrease the incoherent neutron scattering. The pD value was determined as 0.4 plus the pH meter read-out. Data of low concentrated (3–5 mg/mL) protein solutions that show no signs of intermolecular interactions, nor aggregates was used to characterize the form of the protein molecule in each folding/denaturation state (see Fig. 3). All measurements in this study were performed at 10 °C to minimize the risk of aggregation. The scattering curve of apoMb at pD 6 is well described by a generalized Guinier model^29^, $I(q)\ =\ A{e}^{(-{R}_{g}^{2}{q}^{2})/(3-\alpha )}$, where *Rg* is the radius of gyration and *α* a parameter describing the three-dimensional form of the protein. The model is valid in the range: *qRg* < 1.3. With *α* = 0, the protein is a spheroid with *Rg* = 1.5 nm. With a hydrodynamic radius *R*_*H*_ of approximately 2 nm determined by dynamic light scattering (DLS), this state has a high degree of compactness: *R*_*H*_/*Rg* = 1.32 (^30^ and the references therein). The theoretical limit of a solid sphere is given by *R*_*H*_/*Rg* = (5/3)^0.5^ = 1.29, while the average for a random-coil polymer (or a polymer in *θ*-solvent) gives a ratio of 0.65^31^.

The measured SANS curves of the partially and completely unfolded proteins are well described by the polymer with excluded volume model^32,33^ (See Table 1 and Fig. 3). This analytical model was used to describe various polymer systems^34^. Whereas a Gaussian polymer chain has orientationally uncorrelated links between the beads and the length of these segments follows a Gaussian probability distribution, this model considers excluded volume effects too, reflected by the excluded volume parameter *ν*. This is related to the Porod exponent *m* through *ν* = 1/*m* and also known as critical exponent. The statistical segment length of the polymer chain, also known as Kuhn length *l*, and the degree of polymerization *n* can be extracted from the formula ${R}_{g}^{2}\ =\ {l}^{2}{n}^{2\nu }/[(2\nu +1)(2\nu +2)]$.

**Table 1:** Form factor fit results and their comparison with the hydrodynamic radius. *R*_*H*_/*Rg* reflects the compactness of the species. pD 4 is the most compact of the polymer-alike species, approaching the theoretical limit of a sphere of 1.29. The Kuhn length *l* is determined under the assumption that the unfolded protein consists of 20 identical beads (N = 20, having on average 7 amino acids per bead for monomeric apoMb). The errors reported for *Rg* and the Porod exponent are fitting errors, the ones for *R*_*H*_ are the standard deviation of the size distribution obtained using the CONTIN algorithm. The reported errors do not account for any systematic sources of error.

	*R*_*g*_/Å	*ν*	*m*	*R*_*H*_/Å	*R*_*H*_/*Rg*	*l*/Å
pD 2	26.7 ± 3.5	0.55	1.83 ± 0.35	18.0 ± 2.6	0.67	13.6
pD 4	25.4 ± 0.7	0.46	2.17 ± 0.02	30.0 ± 5.6	1.18	15.1
GdmCl	70.2 ± 0.6	0.64	1.56 ± 0.01	56.7 ± 4.8	0.81	28.2
pD 6	14.8 ± 0.1	—	—	19.6 ± 2.5	1.32	—

The compactness of a polymer is also related to the excluded volume parameter *ν*^30^. Applying the polymer with excluded volume model to the present denaturated protein structures is appropriate, given that the theory behind it is validated in practice by several techniques. In a simple picture, the denaturation by acid occurs because the amino acid side chains become protonated and repel each other, destabilizing the secondary structure elements. ApoMb at pD 4, the molten globule state with 30% content of secondary structure elements, is more compact (*R*_*H*_/*Rg* = 1.18 and *ν* = 0.46) than apoMb at pD 2 (4% content of secondary structure elements, *R*_*H*_/*Rg* = 0.67, *ν* = 0.55). Denaturation by GdmCl occurs through a slightly different process: some of the amino acid units become protonated (pH meter read-out for the buffer of the GdmCl-denaturated protein is 4.5), and the guanidium hydrochloride molecules interact with the protein chain, leading to an expansion of the unfolded molecule^35–37^. This is reflected in our data: larger *Rg* and *R*_*H*_ values, and also less compactness compared to the other unfolded states: *ν* = 0.64. Similar to the apoMb unfolded state of urea investigated by Eliezer *et al*.^38^, this could be a mixture of monomer and dimer. In other words, apoMb at pD 6 is a typical globular protein, whereas the partially and completely acid-unfolded, apoMb at pD 4 and pD 2 are more compact than the denaturant unfolded state. The GdmCl-denaturated state has a larger size and lacks compactness. ApoMb at pD 2 has the typical *R*_*H*_/*Rg* value for a polymer in good solvent^39,40^ and the typical *ν*-value for a chain with excluded volume interactions^41^.

The structure factor, which is concentration-dependent, is obtained by dividing the scattering curve of the concentrated solution by the form factor (see SI). The data is smoothed and averaged to remove the noise. Whereas the form factor describes the shape of a molecule in solution, the structure factor characterizes the interaction between these molecules. The structure factor is needed in order to correct the dynamics data reported later. Intermolecular interactions are well described in the case of apoMb by a mean spherical approximation (MSA) structure factor^42,43^, originally developed for macro-ion solutions. The model was implemented using the python package Jscatter^44^, an adaptation of the original Fortran code^45^. ApoMb at pD 6 is closer to its isoelectric point (estimated by ExPASy^46^ to lie at 7.20), therefore there is only a slight difference between the number of positively and negatively charged residues. The charge on the surface is not distributed uniformly and the monomers attract each other (the structure factor is larger than 1 in the low q-regime). The curve has its minimum at q = 0.07 Å^−1^, suggesting that monomers start to interact with each other at a typical distance of 2*π*/*q* = 90 Å. A radius of gyration of 17 Å (closely lying to the one obtained by fitting the form factor) and a screening length of 30 Å are obtained by fitting the MSA model. In comparison to apoMb at pD 6, the structure factors of the solutions of apoMb denaturated by acid and GdmCl show that the monomers repel each other. This repelling can be attributed to the charge state (Fit results are available in SI).

### Dynamical properties

Neutron Spin-Echo Spectroscopy (NSE,) measures temporal and spatial correlations between different scattering particles and from internal motions in the particles resulting in the normalized intermediate scattering function (ISF) *S*(*q*, *t*)/*S*(*q*, 0). ISF can be investigated for each q-value: either for its initial slope or as a stretched exponential (Kohlrausch-Williams-Watts). Alternatively, the data can be modelled simultaneously for all q values according to polymer models.

#### Investigation of the spectra initial slope

From its initial slope, the effective diffusion coefficient *D*_1_ is obtained *S*(*q*, *t*)/*S*(*q*, 0) = *Aexp*(− *D*_1_*t* − *D*_2_*t*^2^) (see SI). According to the de Gennes^40^ and Doi^22^ theory, the overlap concentration c^*^ = *M*/(*N*_*A*_4*πRg*^3^/3) is the border between the diluted and the semidiluted regime of a polymer solution. ApoMb has a molecular weight of M = 16951 g/mol (the molecular weight of myoglobin of which the heme group weight is subtracted), and for *Rg* = 2 nm, the calculated value for the overlap concentration is c^*^ = 840 g/L. At 30 mg/mL, the solution is significantly below the overlap concentration, thus it can be treated as a dilute solution. With an assumed *Rg* of 3 nm the overlap concentration would be c^*^ = 249 g/L, still one order of magnitude larger than the maximum protein concentration used in the experiments presented here. However, this dilution classification is derived for polymer systems and does not account for any surface charge or forces between the protein molecules. Empirically, it was shown that intermolecular interactions and the solvent mediated interactions have to be considered as well^47^. Intermolecular interactions are represented by the structure factor. Solvent-mediated interactions are represented by the hydrodynamic function *H*(*c*, *q*), which can be approximated as a q-independent constant, given that its value in the low q regime is close to its value in the high q-regime. At low q-values, *H*_*c,q0*_ = *D*_*c*_*S*_*q*0_/*D*_0_, where *D*_0_ is the extrapolated diffusion constant at infinite dilution, *D*_*c*_ is the diffusion coefficient at concentration *c* measured by DLS, and S(q = 0.026 nm^−1^) is the value of the structure factor at the DLS-specific q-value. At large q-values, the hydrodynamic functions *H*_*c,qL*_ = 0 can be approximated as the ratio between the measured viscosity of the concentrated and diluted protein solution, *η*_*conc*_ and *η*_*c*=0_, respectively. For these solutions of apoMb, the values of *H*_*c*,*q*0_ and *H*_*c*,*qL*_ are close to each other (see Fig. 3) and we assume that the hydrodynamic functions are constant in the q-range of interest.

**Figure 4:**
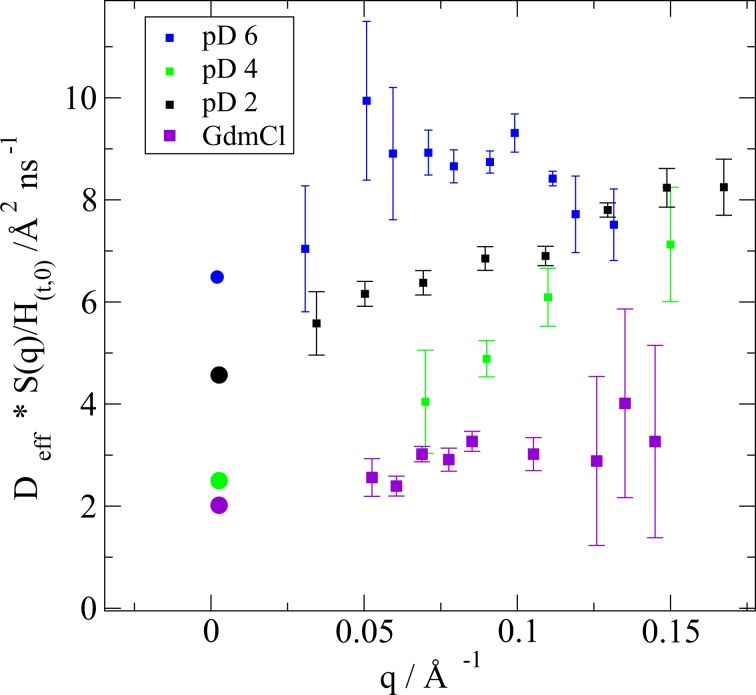
Effective diffusion coefficients obtained from the NSE spectra and corrected for the structure factor and the hydrodynamic function (squares) *D*_*eff*_ of apoMb at pD 4, pD 2 and denaturated by GdmCl has a linear q-dependence, whereas of the apoMb at pD 6 has a more complex dependence. The dots mark the translational diffusion coefficients obtained by DLS.

Thereby, the effective diffusion coefficients *D*_*eff*_ for the protein monomers are obtained: *D*_*eff*_(*q*) = *D*_1_(*q*)*S*(*c*, *q*)/*H*_*c*,*q*0_ (see Fig. 4). They comprise information on translational diffusion, rotational diffusion and internal dynamics of the single molecule. In a good approximation, these motions can be decoupled^18^. For apoMb denaturated by acid (at pD 2 and pD 4) and by GdmCl, *D*_*eff*_ has a linear dependence on q which is specific for the Zimm regime of local chain relaxations^48^, whereas for apoMb at pD 6 the value of *D*_*eff*_ has a non-linear dependence on q (see Fig. 4).

The dynamics of the mostly folded protein apoMb at pD 6 deviates from the dynamics of the more denatured protein solutions. It is therefore discussed in the following paragraphs. At first, translational and rotational diffusion can be determined in the rigid-body approximation, directly from pdb structures using HYDROPRO^49^. ApoMb at pD 6 resembles the native structure of myoglobin. Given that there are no available pdb structures of the heme-free forms, and motivated by the work of Stadler *et al*.^50^, proving that myoglobin and apoMb at pD 6 have similar characteristics in solution, the crystal structure of myoglobin (pdb ID: 2v1k) was used for the calculation. For *T* = 283.15 K, *η* = 1.67 mPas, *ϕ* = 0.720 cm^3^/g (solute partial specific volume) and *ρ* = 1 g/cm^3^ (solution density), the 9x9 diffusion matrix *D* is obtained, that comprises the translational and the rotational diffusion matrices, whose traces are the translational diffusion coefficient 5.96 Å^2^/ns and the rotational diffusion coefficient of 9.83 *μ*s^−1^.

**Figure 5:**
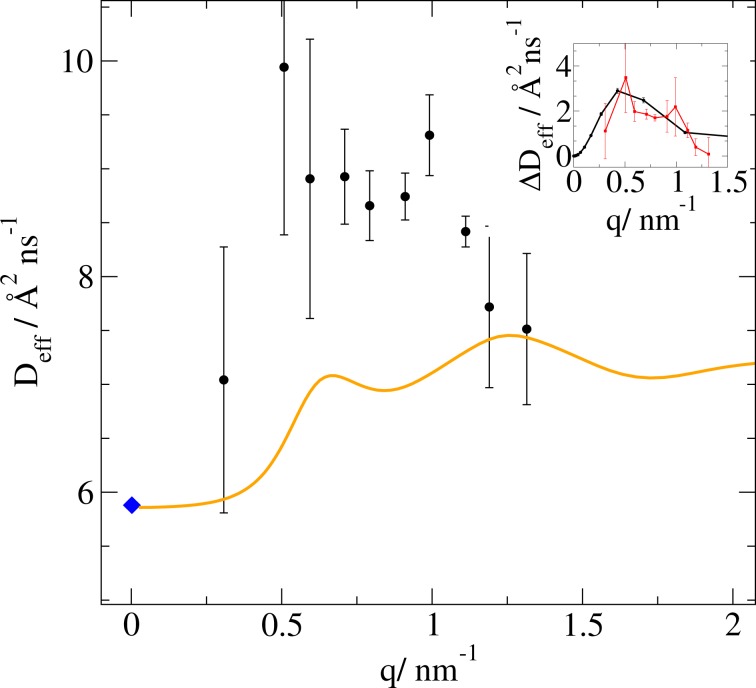
Effective diffusion coefficients of apoMb at pD 6. The blue diamond is the translational diffusion obtained by DLS, the orange curve the *D*_*trans*−*rot*_ function calculated for 2v1k.pdb. *Inset*: The difference between the NSE data and the *D*_*trans*−*rot*_ (red data points) can be attributed to the first non-trivial normal mode of the structure (black line), which corresponds to the opening of two helices to allow the acces to the heme-group.

The q-dependency of the coupled rotational and translational diffusion is obtained from the coordinates of the amino acids in the protein $\overrightarrow{r}$, their individual neutron scattering length *b*, the form factor *F*(*q*), and the diffusion matrix obtained above, using the formula:

1 $${D}_{0}(q)\ =\ \frac{1}{{q}^{2}F(q)}\sum _{j,k}\left\langle {b}_{j}exp\left(-i\overrightarrow{q}\overrightarrow{{r}_{j}}\right)\left(\begin{array}{c}q\\ \overrightarrow{q}\times \overrightarrow{{r}_{j}}\end{array}\right){\bf{D}}\left(\begin{array}{c}\overrightarrow{q}\\ \overrightarrow{q}\times \overrightarrow{{r}_{k}}\end{array}\right){b}_{k}exp(-i\overrightarrow{q}\overrightarrow{{r}_{k}})\right\rangle $$

which is derived by Ortega *et al*.^47^. The brackets represent the ensemble average over the remaining variables. While the integration over the position space for the single particle is 1, the orientation average can be replaced by an averaging over q-space. The exchange occurring between the protons at the protein surface and the solvent is also considered. The calculated *D*_0_(*q*) values are shown in Fig. 5 together with the experimentally derived *D*_*eff*_(*q*) values. As can be seen in Fig. 5, the difference *ΔD*_*eff*_(*q*) = *D*_*eff*_(*q*) − *D*_0_(*q*) between the measured NSE data points and the calculated *D*_*trans*−*rot*_ accounts for approximately 20% of the total dynamics and can be due to internal *α*-helices movements or other internal dynamics processes. We performed a normal mode analysis using the MMTK package^51–53^. We determined the effective diffusion specific for the first non-trivial mode, mode number 7, as following:

2 $${D}_{eff}^{\alpha =7}(q)\ =\ \frac{{\lambda }_{\alpha =7}{k}_{\alpha =7}}{{q}^{2}F(q)}\left\langle \sum _{j,k}{b}_{j}{b}_{k}exp\left(-i\overrightarrow{q}\cdot (\overrightarrow{{r}_{j}}-\overrightarrow{{r}_{k}})(\overrightarrow{q}\cdot {\overrightarrow{e}}_{k}^{\alpha =7})(\overrightarrow{q}\cdot {\overrightarrow{e}}_{l}^{\alpha =7})\right)\right\rangle $$

where ${\overrightarrow{e}}^{\alpha =7}$ is the normal mode eigenvector, ${\omega }_{\alpha =7}^{2}$ the eigenvalue of the corresponding dynamical matrix, *λ*_*α*=7_ the mode-dependent relaxation rate, and ${k}_{\alpha =7}\ =\ \frac{{k}_{B}T}{\bar{m}{\omega }_{\alpha =7}^{2}}$ the mode-dependent amplitude with average mass $\bar{m}$ at the temperature *T*. We observe that the diffusion coefficient of the first non-trivial normal mode of the pdb structure 2v1k, describing the movement of the *α*-helices which allows the access to the heme group, has a similar dependence of the diffusion coefficient on q, see inset of Fig. 5.

#### Investigation of the spectra using stretched exponential functions

Another common practice in the NSE data evaluation is modeling using a stretched exponential function, characteristic for relaxation processes: $S(q,t)/S(q,0)\ =\ Aexp[-({D}^{^{\prime} }{q}^{2}{t}^{\beta })]$. The stretching exponent *β* for apoMb at pD 6 is on average for all q-dependent data sets 0.9, a value close to 1, so that the protein is seen rather as a point, where translational diffusion dominates, and the internal dynamics is small in comparison to it (about 20%). In contrast, for the pD 2, pD 4 and GdmCl denaturated forms of apoMb the average values of the stretching exponent *β* are: 0.80, 0.85 and 0.81, respectively, approaching the Zimm prediction for Gaussian polymers in solution of 0.85, which indicates the presence of a broad distribution of internal diffusive motions^48^ (see SI).

#### Modelling with polymer models

Dynamics of such polymer-like systems can be well described using Zimm dynamics and a model derived from it, the Zimm model with internal friction (ZIF). The diffusive motions of a finite chain consisting of *N* beads with Kuhn length *l* are characterized by the Langevin equation. Including hydrodynamic interactions and the excluded volume effect, the equation can be transformed into normal coordinates and it leads to the dynamic structure factor:

3 $$\begin{array}{ccc}I(q,t) & = & \frac{exp[-{q}^{2}{D}_{0}\frac{H(c,q)}{S(c,q)}t]}{N}{\Sigma }_{n,m}^{N}exp(\frac{-{q}^{2}B(n,m,t)}{6})\\ B(n,m,t) & = & {(n-m)}^{2\nu }{l}^{2}+\frac{4{R}_{E}^{2}}{{\pi }^{2}}{\Sigma }_{p=1}^{{p}_{max}}\frac{A(p)}{{p}^{2\nu +1}}\\  &  & \times cos(\frac{\pi pn}{N})cos(\frac{\pi pm}{N})[1-exp(-\frac{t}{{\tau }_{p}})]\end{array}$$

*τ*_*p*_ is the relaxation time characteristic for the normal mode *p*, with *η* the solvent viscosity, and *ν* the critical exponent, *k*_*B*_ the Boltzmann’s constant, *T* the temperature. *R*_*E*_ is the end-to-end distance of the polymer chain ${R}_{E}\ =\ \sqrt{(2\nu +1)(2\nu +2)}Rg$. In the exponent of the first term of equation 3 one can find the hydrodynamic function H(c,q) mentioned earlier devided by the structure factor S(c,q). In the Zimm and ZIF models, the normal modes have all the same amplitude: *A*(*p*) = 1. Internal friction reflects the intrinsic resistance of a polymer to changes in its conformation and occurs due to dihedral angle rotational barriers, hydrogen bonding or intrachain collisions. As opposed to the Zimm model, the ZIF model incorporates the internal friction of the polymer chain as a resistive spring installed in parallel to the entropic spring connecting the beads. By solving the Langevin equation, a mode independent relaxation time *τ*_*intern*_ is obtained. It is added to each Zimm mode *τ*_*p*_ so that *τ*_*pZIF*_ = *τ*_*p*_ + *τ*_*intern*_. This way, in the ZIF model the higher frequency normal modes of the Zimm model are damped.

**Figure 6:**
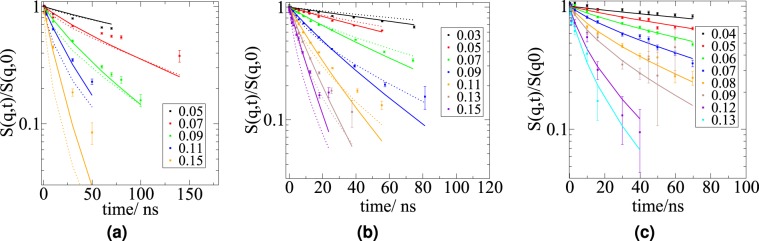
NSE spectra, simulations using the Zimm model (dotted lines) and ZIF fits (lines) of apoMb at pD 4 (**a**), pD 2 (**b**) and denaturated by GdmCl (**c**). In the legend the q-values are reported in Å^−1^.

The NSE spectra can be simulated based on the equation defining *I*(*q*, *t*). Using the information on the translational diffusion from DLS, on the viscosity (from direct measurements), on the hydrodynamic function (see Table 2) and on the critical exponent *ν* and *Rg* obtained from the SANS data (see Table 1), the simulation can be performed. In Fig. 6a,b, the dotted lines are simulated NSE spectra of apoMb pD 4 and pD 2 using the Zimm model, under the assumption that the polymer consists of 20 beads. The simulation reproduces the spectra well, but the large q-values and the longer Fourier-times are not described properly by the Zimm model. Without any knowledge on the internal friction time, the ZIF model was fitted simultaneously for all q for each sample, having only *D* and *τ*_*intern*_ as free parameters. The fit results are presented in Table 3. The values obtained for the center of mass diffusion coefficients *D* are comparable within error bars with the ones obtained via DLS measurements for both pD 2 and pD 4. Although apoMb at pD 4 is a molten globule and has a significantly higher content of secondary structure elements, its dynamics can still be understood similarly to the one of the totally unfolded state. The whole structure needs a similar time to relax (*t*_*Zimm*_) and both protein states experience a similar internal friction(*τ*_*intern*_). However, for apoMb at pD 4, the ZIF model deviates significantly from the experimental NSE spectra at longer Fourier times, especially for the ISF at q = 0.07 Å^−1^, which is reflected in the larger *χ*^2^ value. This could be because the model does not account for any residual secondary structure content. An interpretation of the experimental NSE data might be achieved by coarse-grained computer simulations, which are out of the scope of the present manuscript. We refer here to future studies to clarify that aspect.

**Table 2:** Values of the hydrodynamic functions in a low (*H*_*c*,*q*0_) and large q-regime (*H*_*c*,*qL*_) determined by different methods. The SANS, DLS and viscosity measurements were performed at 283 K. The viscosity value *η*_*conc*_ of the apoMb pD 2 solution with the highest concentration could not be determined accurately.

	*S*_*q*0_	*D*_*conc*_/Å^2^ ns^−1^	*D*_0_/Å^2^ ns^−1^	*H*_*c*,*q*0_	*η*_*conc*_/mPas	*η*_*c*=0_/mPas	*H*_*c*,*qL*_
pD 2	0.485	6.0	3.8	0.103	*	1.74	—
pD 4	0.436	1.4	2.5	0.244	5.01	1.72	0.344
GdmCl	0.615	2.3	2.0	0.708	2.83	1.97	0.696
pD 6	1.163	6.1	8.2	0.863	2.2	1.70	0.773

**Table 3:** Fit parameters (*ν*, *η*, *l*) and results (*D*, *t*_*Zimm*_, *τ*_*intern*_) for the ZIF model. ApoMb at pD 4 and pD 2 have a comparable Zimm and internal friction relaxation time. ApoMb denaturated by GdmCl has almost the double Zimm relaxation time and no internal friction time. Additional parameters that were used for fitting: T = 283.15 K (corresponding to the temperature the experiments were performed at), N = 20 beads, all 20 modes contribute to relaxation.

	*ν*	*η*/*mPas*	*D*/Å^2^ ns^−1^	*l*/nm	*t*_*Zimm*_/ns	*τ*_*intern*_/ns	*χ*^2^
pD2	0.55	1.7	3.0	1.36	49.66	50.89	9.37
pD4	0.46	1.7	1.7	1.51	42.51	49.63	34.12
GdmCl	0.64	1.9	1.2	1.34	83.6	0	2.27

In contrast to these two, apoMb denaturated by GdmCl has almost double the Zimm relaxation time and no internal friction time is observed (see Fig. 6c). Dynamics of denatured apoMb can be described very well using the Zimm model only. This supports the mechanism of denaturation described by Heyda *et al*. and Huerta *et al*.^35,37^: GdmCl increases the solubility of hydrophobic residues and the local energetic barriers are lowered. The trends observed on intrinsically disordered proteins (IDPs) denaturated states in different concentrations of GdmCl^36^ are confirmed. Several studies including the Zimm model support the idea that the centre of mass diffusion coefficient of the protein scales with the chain length, or with the bead number, according to $1/\sqrt{N}$^6,26,36^. These studies are performed on proteins where the chain length is varied, which is not the case for the present work. ApoMb, which always consists of the same number of amino acids, independently of its folding state. The bead number should not be confused with the chain length. The choice of the beads number when the protein is considered a polymer is arbitrary, but even when we increase the beads number, having less than 7 amino-acids per bead, the ZIF model does not change its validity (see Supplementary Information).

Further polymer models (Zimm with damping of the mode amplitudes^21^, compacted Zimm with internal friction^26^, the Zimm analogues of the Rouse with non-local interactions and of the Rouse with anharmonic potentials^54^ have been considered to interpret this data, but none leads to better results. Some studies claiming that internal friction does not play a role are performed on smaller proteins^28^ or the solvent viscosity is varied significantly^55^. In those cases, the friction with the solvent, and not the internal friction may be the dominant dissipation mechanism. For the data presented in this work for apoMb at pD 2 and pD 4, the ZIF model is the best fit.

## Discussion

By comparing two different denaturation ways, we could gain insights on the denaturant effect on the structure and dynamics of the model system apomyoglobin. Both ways started from the native-alike form apoMb at pD 6. The protein in this folding state resembles many structural features of the holoprotein and its dynamics shows internal collective modes, which are no longer seen in any other unfolded states investigated (see Fig. 1). Its internal dynamics, accounting for less than 20% of the total dynamics of the protein is of biological relevance: the *α*-helices perform this movement to incorporate the heme group in the process of the protein synthesis^56^.

In case of the acid denaturation, apoMb at pD 4 has a high content of secondary structure elements, observed by CD spectroscopy and SANS. However, its dynamics can if it all be described by the same polymer model (ZIF) as the dynamics of the acid unfolded state, apoMb at pD 2 (see Fig. 1B,C,G). Although similar Zimm relaxation and internal friction times are obtained, the data is not as perfectly modelled. The GdmCl unfolded apoMb does not show internal friction, suggesting that this denaturant is screening the protein chains, reducing the interaction between them (see Fig. 1D,F). The observations of Zheng *et al*.^56^, Borgia *et al*.^36^ and Samanta *et al*.^26^ on IDPs are confirmed also for apoMb: internal friction is larger with considerable increase of protein compactness.

Previous QENS experiments showed that molecular dynamics on the faster ps to ns time-scale are similar between apoMb at pD 2 and apoMb at pD 4, but differ significantly from apoMb at pD 6^57^. That dynamic picture is corroborated here by NSE for slower collective dynamics as well. The first folding step in apoMb does not have a significant effect on collective internal dynamics. A fundamental change in the physical nature of the dynamics of Mb due to protein folding occurs only by the following folding step into the native state, where the heme-pocket is formed. By comparing the internal friction in apoMb at pD 4 with that of an IDP with a similar content of secondary structure^20^, we see that internal friction dominates the Zimm mode spectrum even stronger for the IDP than for the apo-Mb at pD 4. This shows that apoMb at pD 4 and apoMb at pD 2 still need to be seen as comparatively soft protein conformations. Therefore, the formation of the G and H helices in the apoMb at pD 4 state is not that important for the motions seen by NSE. Motions in apoMb at pD 2 and pD 4 are rather influenced by the transient formation of secondary structure content. If more information on intermediate states experiencing constant folding/refolding transitions would be available, the dynamics of the denaturated proteins observed by NSE could be modelled as an equilibrium, an average distribution of the intermediate state dynamics. Recent single-molecule techniques allow the observation of such intermediate states^58^, whilst theories such as Zimm-Bragg^59^ claim that chemical unfolding is a multi-state process of a mixture of conformations. To relate the NSE observations with the in depth understanding of the chemical unfolding process of apoMb directly, such experiments and theories would be necessary.

Although proteins are known to adopt their unique structure based on the individuality of their amino acid side chains, coarse grain polymer models can characterize the nanosecond dynamics. In case of the GdmCl denatured apomyoglobin, the protein loses all its protein-like features and behaves like a Zimm polymer. This is mostly due to the binding of GdmCl to the side chains removing their individuality leading to a more polymer like behavior. Moreover, apoMb at pD 2, which could still exhibit hydrogen bonding and some transient elements of secondary structure, loses its protein-like features, but behaves like a non-ideal polymer, with internal friction.

## Methods

### Sample preparation

ApoMb was prepared from horse-heart myoglobin (Sigma-Aldrich) following the butanone method to extract the heme group (as performed in^50^), adapting the method described in^60^), and then refolded by dialysis in 20 mM NaH_2_PO_4_/Na_2_HPO_4_ (Sigma Life Science, >99.5% and Sigma-Aldrich, >99%) pH 7 buffer and distilled water. Before storage in the freezer, the apo-Mb solution was lyophilized. To replace the exchangeable protons by deuterium ions, the freeze-dried apo-Mb powder was dissolved in heavy water (99.9% ^2^H, Sigma-Aldrich), incubated for 1 day, and lyophilized again. The obtained powder was stored at −20 °C. In order to obtain the molten globule state of apoMb the powder was dissolved in ^2^H_2_O and centrifuged to remove the large aggregates. In the supernate solution of concentration 2 mg/mL and pH 6, ^2^HCl 0.1 M (Sigma-Aldrich) was added until the pH-read out value was 3.6 (monitored by pH meter Methrom). This corresponds to a a pD value of 4. The buffer exchanged protein solution was centrifuged (Heracus Instruments) to the final concentrations (Vivaspin 3,000 MWCO concentration units, Sartorius, Göttingen, Germany).

### Circular dichroism (CD)

Circular dichroism was measured on a Jasco J1100 spectropolarimeter (JASCO, Tokyo, Japan), in the range 180–250 nm, with a pitch of 1 nm, a scanning speed of 100 nm/min, and 3 accumulations/measurement. The samples were measured at a concentration of 300 *μ*M in 0.01 cm thick quartz cuvettes under constant nitrogen flow at 10 °C. According to the BeStSel Single Spectrum Analysis^61^, the *α*-helix composition of apoMb, varied as following: pD 6–49%, pD 4–25%, pD 2–4.3%, GdmCl–6%. In case of GdmCl-denaturated solution, only the range 200–240 nm was considered for data analysis because GdmCl absorbs strongly in the range 180–200 nm.

### Dynamic light scattering (DLS)

Dynamic light scattering was measured on a Zetasizer Nano ZS instrument (Malvern Instruments, Malvern, Worcestershire, United Kingdom), that records the 632.8 nm light scattered under 173°. Autocorrelation functions were analyzed by a CONTIN algorithm. The hydrodynamic radius R_H_ was determined according to the Stokes-Einstein equation *R*_*H*_ = *k*_*B*_*T*/(6*πηD*) with the D_2_O viscosity *η* = 1.679 mPa s at 10 °C. The 30 mg/mL solutions of apoMb at pD 4 and apoMb denaturated by GdmCl that were also monitored by *in situ* DLS, during the NSE experiments^62^.

### Small-angle neutron scattering (SANS)

The scattering vector q is defined as *q* = 4*nπ*/*λsin*(*θ*∕2) with the incident neutron wavelength *λ* and the scattering angle *θ*. The investigation of the form and structure factor was performed for apoMb at pD 2 and pD 6 at the instrument KWS-2 at the MLZ in Garching^63^. The *in situ* DLS option at this instrument helped to acquire data that confirmed that the samples did not show considerable aggregation during the neutron measurement. Protein concentrations were 3, 6, 15 and 30 mg/mL. The corresponding buffers, empty cells and references were measured as well. Hellma quartz cells of 1 mm and 2 mm were used for high- and low protein concentrations. The neutron wavelength was set to 4.5 Å, and measurements were performed at 3 detector positions: 2, 8 and 20 m. All measurements have been performed at 10 °C. For the low-concentrated solutions, the background-corrected intensities were linearly extrapolated to infinite dilution to extract the form factor per unit mass. The measured SANS curve of apoMb at pD 2, pD 4 and GdmCl are well-described by a polymer with excluded volume model, while apoMb at pD 6 is globular, thus the corresponding SANS curve is described by a Guinier model. By dividing the SANS curve of the highest concentration by the one at the lowest, the structure factor was obtained.

### Neutron spin-echo spectroscopy (NSE)

Solutions of apoMb at pD 2 were investigated at the instrument SNS-NSE, the neutron spin echo spectrometer at the Oak Ridge National Laboratory, Oak Rigde, Tennessee, USA^64^. It is a time-of-flight instrument: the Larmor precession of the neutron spin in a preparation zone with magnetic field before the sample encodes the individual velocities of the incoming neutrons into a precession angle. The other samples were measured at J-NSE “Phoenix” at MLZ, Garching^65^. The instrument covers a Q-range of 0.03–1.0 Å^−1^, reaching Fourier Times of 250–90 ns using 12 and 8 Å neutrons. In the experiments presented here, a Q-range of 0.03–0.15 Å^−1^ was explored using using 12 and 8 Å neutrons. A graphite powder sample was measured as a scattering reference, followed by the protein sample and the buffer solution. All measurements were performed at 10 °C. NSE data evaluation was performed with the data reduction software DrSPINE^66,67^.

### Viscosimetry

The viscosity of all protein solutions and buffers was measured at 10 °C using a rolling-ball viscometer Lovis 2000 M/ME. Each measurement was performed 3 times and the average value was reported.

### UV/VIS spectroscopy

The sample absorption in a cell with a path-length of 0.1 mm (Hellma, Germany) was measured using UV/VIS Spectroscopy (Cary 300). For the very low concentrations (<1 mg/mL), a 5 mm thick quartz Hellma cell was used. The concentrations were determined from the absorption values using the molar extinction coefficient *ϵ*_280*nm*_ = 13980 M^−1^ cm^−1^ calculated from the amino acid sequence (ExPASy^46^).

## Supplementary information

Supplementary Information.
