# Supplementary material for: Transition between protein-like and polymer-like dynamic behavior: Internal friction in unfolded apomyoglobin depends on denaturing conditions
Source: Sci Rep. 2020 Jan 31;10:1570. doi: 10.1038/s41598-020-57775-4 (PMC6994677; doi:10.1038/s41598-020-57775-4)
Supplement: Supplementary file 1 — Supplementary Information. [file 41598_2020_57775_MOESM1_ESM.pdf]

Supplementary Information to: Transition between protein-like and polymer-like dynamic behavior: Internal friction in unfolded apomyoglobin depends on denaturing conditions

Livia Balacescu[1,2], Tobias E. Schrader[1,*], Aurel Radulescu[1], Piotr Zolnierczuk[3], Olaf Holderer[1], Stefano Pasini[1], Jörg Fitter[2,4], and Andreas M. Stadler[5,6]

[1]Forschungszentrum Jülich GmbH, Jülich Centre for Neutron Science (JCNS) at Heinz Maier-Leibnitz Zentrum (MLZ), Lichtenbergstr. 1, 85748 Garching
[2]I. Physikalisches Institut (IA), AG Biophysik, RWTH Aachen
[3]Forschungszentrum Jülich GmbH, Jülich Centre for Neutron Science (JCNS) Outstation at Spallation Neutron Source (SNS), Oak Ridge, TN 37831, USA
[4]Forschungszentrum Jülich GmbH, Institute for Complex Systems (ICS-5), 52425 Jülich
[5]Institute of Physical Chemistry, RWTH Aachen University, Landoltweg 2, 52056, Aachen, Germany
[6]Forschungszentrum Jülich GmbH, Jülich Centre for Neutron Science (JCNS-1) and Institute for Complex Systems (ICS-1), 52425 Jülich
*t.schrader@fz-juelich.de

January 22, 2020

Form Factors

The partially and completely unfolded proteins (apoMb at pD 2 and pD 4 and denaturated by GdmCl) are well described by the polymer with excluded volume model, characterized by two parameters: the radius of gyration and the Flory exponent ν. ApoMb at pD 6 cannot be characterized using polymer models, but by the Guinier model, typical for a globular protein.

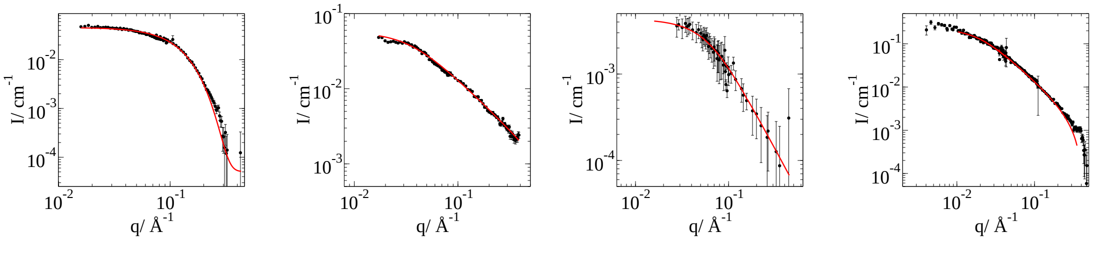

(a) **(b)** **(c)** **(d)**

Figure 1. 5 mg/mL concentrated protein solutions of pD 6 (a), pD 4 (b) and pD 2 (c) and GdmCl (d) denaturated apoMb: background-subtracted SANS, interpreted using the Guinier [1] and the polymer with excluded volume models [2, 3], respectively.

Structure Factors

Intermolecular interactions are well described in the case of apoMb by a mean spherical approximation (MSA) structure factor [6, 7], originally developed for macro-ion solutions. This model accounts for several parameters: the radius of the object R, the solution molarity, the screening length *scl* and the contact potential in k_T units γ.

	R/ nm	molarity/ mM	scl/nm	γ
pD 6	1.77	3.285 e^{-4}	3.027	-4.5
pD 4	4.46	0.30253	1.495	542.4
pD 2	2.50	0.4732	2.196	12.64
GdmCl	2.80	0.10228	2.523	107.9

Table 1. Fit results for the structure factor using the MSA model

The contact potential γ depends on the electronic surface charge z_m, on the free permittivity e_0, the dielectric constant e and on the inverse screening length k=1/scl: $\gamma = z_m/[\pi e_0 e R(2+kR)]$.

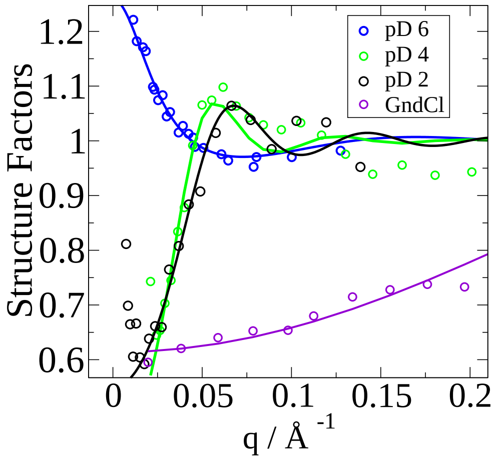

Figure 2. Structure factors of apoMb at pD 6, pD 4, pD 2 and denaturated by GdmCl and the MSA fitting.

NSE Spectra fitting

Stretched exponential fitting

Most of the relaxation processes in soft materials cannot be described by a single exponential decay. They are usually strongly stretched and can be described in the time domain by the Kohlrausch–Williams–Watts (KWW) relationship: $exp[(t/\tau)^{\beta}]$, where τ is the characteristic relaxation time and β is the stretching parameter. NSE is the only inelastic neutron technique which acquires the spectra in time domain, therefore the easiest interpretation of protein dynamics is performed through a KWW function: $S(q,t)/S(q,0) = Aexp[-(D'q^2t^{\beta})]$ (see Fig 3).

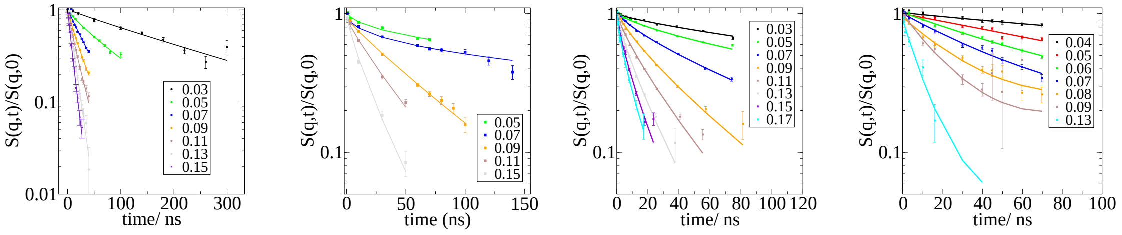

(a) (b) (c) (d)

Figure 3. NSE Spectra of apoMb solutions (30 mg/mL) of pD 6 (3a), pD 4 (3b) and pD 2 (3c) and GdmCl (3d) denaturated apoMb fitted using the stretched exponential function.

Fitting of the spectra initial slope

From the spectra initial slope, the effective diffusion coefficient is obtained (D_1 from $I(q,t)/I(q,0) = Aexp(-D_1t - D_2t^2)$) (see Fig 4).

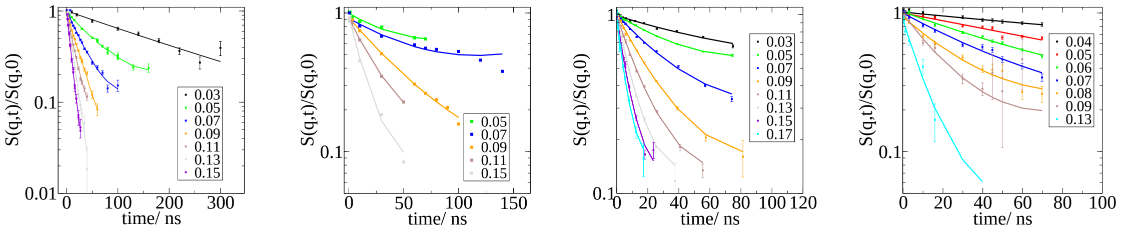

(a) (b) (c) (d)

Figure 4. NSE Spectra of apoMb solutions (30 mg/mL) of pD 6 (4a), pD 4 (4b) and pD 2 (4c) and GdmCl (4d) denaturated apoMb fitted to obtain the initial slope.

Zimm, Zimm with internal friction and Zimm with mode damping

The data sets of apoMb at pD 4 and pD 2 were interpreted using the Zimm and Zimm with mode damping models. The fitting of the Zimm model was performed to get a more precise value for the diffusion coefficient, in comparison to the ones obtained by DLS. The value is then obtained by considering the contribution of the hydrodynamic function and the structure factor. The DLS values are considerably higher: for apoMb at pD 2, D_{DLS}=3.8 Å^2/ns and for apoMb at pD 4, D_{DLS}=2.5 Å^2/ns, while from the Zimm fit, for apoMb at pD 2, D=2.2 Å^2/ns, and for apoMb at pD 4, D=1.3 Å^2/ns.
To recall, Zimm with mode damping is an extension of the Zimm model, where topological confinement is taken into account [4, 5]. The temporary formation of α-helix which is specific for the molten globule state could lead to such a topological confinement in the polymer structure. This topological confinement reduces the amplitudes of the larger normal modes. Empirically, a Fermi function was chosen to describe the damping of the mode amplitudes: $A(p) = 1 - [1/exp((p-p_0)/\sigma)+1]$.

For the data set of apoMb at pD 4, this model seems to match the large q-values better than the ZIF model, especially for the larger Fourier times (see Fig. 5 and Table 3). However, this model leads to a χ^2 value which is slightly above the value obtained by fitting the ZIF model, due to the spectra points at low q-values and short Fourier times, which have a larger weight in the fitting. A similar result is obtained for the NSE spectra of apoMb at pD 2. Therefore, we cannot conclude based on these data sets, that any topological confinement can be observed or modelled.

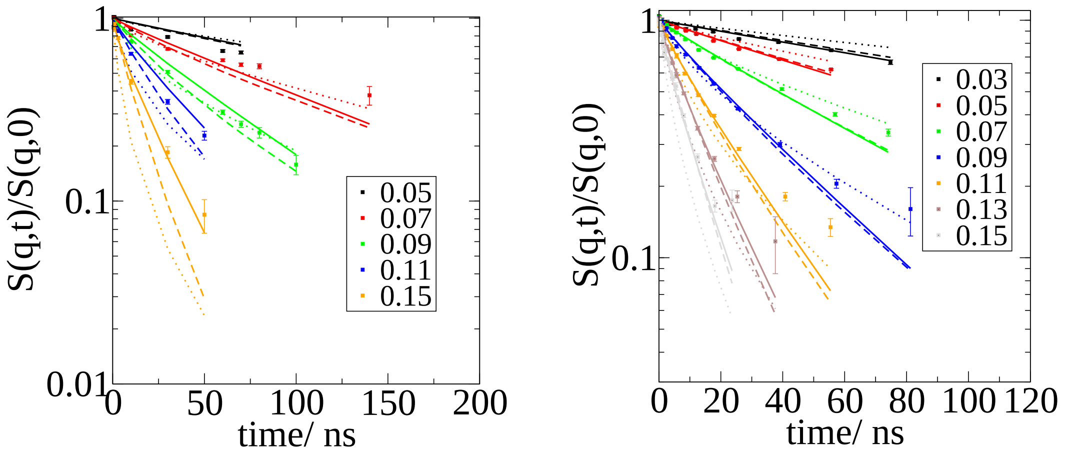

(a) **(b)**

Figure 5. NSE spectra of apoMb at pD of apoMb at pD 4 (5a) and pD 2 (6b), fits using the Zimm model (dotted lines), fits of the Zimm with internal friction- ZIF model (dashed lines) and fits of the Zimm with reduced mode amplitudes model (straight lines). In the legend the q-values are reported in $Å^{-1}$

	Model	ν	η/ mPas	l/ nm	D/ $Å^2$/ns	t_{ZIMM}/ns	t_{intern}/ns	p_0	σ	χ^2
pD 2	Zimm	0.55	1.7	1.36	2.16±0.10	50.54	-	-	-	56.74
	ZIF	0.55	1.7	1.36	3.03±0.08	50.54	50.35±6.55	-	-	11.21
	ZMD	0.55	1.7	1.36	3.38±0.09	50.54	-	6	5	7.94
pD 4	Zimm	0.46	1.7	1.5	1.35±0.15	42.5	-	-	-	52.55
	ZIF	0.46	1.7	1.5	1.73±0.18	42.5	49.64±22.34	-	-	34.12
	ZMD	0.46	1.7	1.5	1.90±0.19	43.6	-	10	5	39.45

Table 2. Fit results for the Zimm model, Zimm with internal friction (ZIF) and Zimm with mode damping (ZMD) models perfomed simultaneously for all q-values. T=283.15 K, 20 modes, 20 beads, $p_{max} = 20$

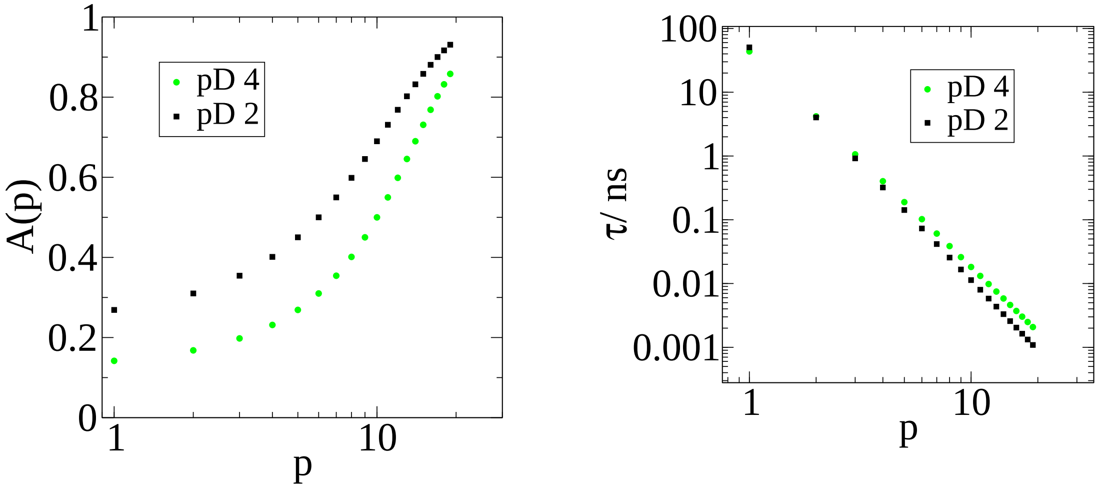

(a) **(b)**

Figure 6. (left) Mode amplitude as function of mode number p. (right) Characteristic Zimm times $\tau(p)$ for each mode.

Bead number dependence

When the NSE data is fitted using models from polymer theory, the choice of bead number is free. For the data presented in this paper and its interpretation, the "polymer protein" consists of N=20, which corresponds to an average of 7 amino acids per bead for monomeric apoMb. Hereby we present the fit results by applying the ZIF model on the NSE data of the apoMb at pD 2. If the beads number N is varied from 20 to 140 units and the Kuhn length is calculated accordingly, there are no significant changes in the obtained values for the center of mass diffusion coefficient, Zimm relaxation time, internal friction time, nor fitting accuracy, reflected by χ^2. All the results are shown in Figure 7.

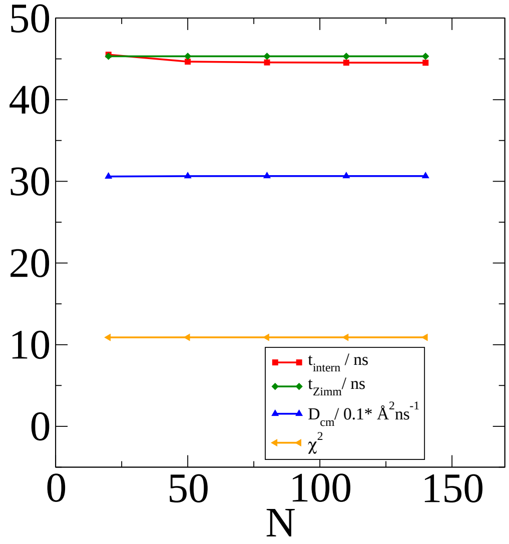

Figure 7. Parameters from the ZIF models (t$_{Zimm}$ and t$_{intern}$ in ns, D$_{cm}$ in 0.1 $^2 ns^{-1}$) as a function of beads number

References

1. B. Hammouda, A new guinier–porod model, J. Appl. Cryst. **43**, 716 (2010) .
2. A.-J. Petrescu, V. Receveur, P. Calmettes, D. Durand, and J. C. Smith, Excluded volume in the configurational distribution of a strongly-denatured protein, Prot. Sci. **7**, 1396 (1998) .
3. A.-J. Petrescu, V. Receveur, P. Calmettes, D. Durand, M. Desmadril, B. Roux, and J. C. Smith, Small-angle neutron scattering by a strongly denatured protein: Analysis using random polymer theory, Biophys. J. **72**, 335 (1997) .
4. C. Mark, O. Holderer, J. Allgaier, E. Hübner, M. Zamponi, A. Radulescu, A. Feoktystov, M. Monkenbusch, N. Jalarvo, and D. Richter, Polymer chain conformation and dynamical confinement in a model one-component nanocomposite, Phys. Rev. Lett. **119**, 047801 (2017) .
5. F. Ameseder, A. Radulescu, O. Holderer, P. Falus, D. Richter, and A. M. Stadler, Relevance of internal friction and structural constraints for the dynamics of denatured bovine serum albumin, J. Phys. Chem. Lett. **9**, 24692473 (2018) .
6. L. Belloni, Colloidal interactions, J. Phys.: Condens. Matter **12**, R549 (2000) .
7. J.-P. Hansen and J. B. Hayter, A rescaled MSA structure factor for dilute charged colloidal dispersions, Molecular Physics: An International Journal at the Interface Between Chemistry and Physics **46**, 651 (1982).
